# Seasonal profile of metal accumulation in the acanthocephalan *Pomphorhynchus laevis*: a valuable tool to study infection dynamics and implications for metal monitoring

**DOI:** 10.1186/s13071-016-1576-4

**Published:** 2016-05-23

**Authors:** Milen Nachev, Bernd Sures

**Affiliations:** Department of Aquatic Ecology and Centre for Water and Environmental Research (ZWU), University of Duisburg-Essen, Universitätsstraße 5, Essen, D-45141 Germany; Department of Zoology, University of Johannesburg, Johannesburg, South Africa

**Keywords:** Metals, Accumulation, *Pomphorhynchus laevis*, *Barbus barbus*, Seasonality, Acanthocephalans

## Abstract

**Background:**

A large number of studies demonstrated that acanthocephalans exhibit a high metal accumulation potential and thus can be used as sensitive accumulation indicators. However, similar to free-living bioindicators, a seasonal variation in metal concentrations in parasites might occur. Accordingly, the influence of seasonality has to be elucidated if parasites should be applied as sentinels.

**Methods:**

In order to assess a possible seasonal profile of element concentrations, the concentrations of As, Cd, Co, Cu, Fe, Mn, Mo, Ni, Pb, V and Zn in the acanthocephalan *Pomphorhynchus laevis* and in its host barbel (*Barbus barbus*) were analysed in a seasonal manner (spring, summer and autumn) using inductively coupled plasma mass spectrometry (ICP-MS).

**Results:**

Five elements (As, Cd, Cu, Pb and Zn) were detected in significantly higher concentrations in the parasites compared to host muscle, intestine and liver. Their levels in *P. laevis* showed a clear seasonal pattern, while the concentrations in the fish tissues remained similar during the year. The highest concentrations in the parasites were found in autumn, followed by spring and summer. Evidence from the literature suggests that this profile coincides with the seasonality of acanthocephalan transmission, as their annual concentration profile reflected the mean individual weight pattern during the year. Parasite infrapopulations in autumn consisted mainly of young worms which are characterised by an accelerated metabolism and a higher surface to volume ratio resulting in higher element concentrations when compared to older worms which are assumed to slow down their metabolism and additionally excrete metals with their eggs.

**Conclusions:**

Based on the available data from the present study and literature, a model is suggested, which visualises the accumulation kinetic of several elements under natural conditions. According to the element accumulation data the lifespan of *P. laevis* in barbel was roughly estimated to range between six and eight months.

## Background

Despite a large number of studies on metal accumulation in different host-parasite systems [1, 2], information about seasonal dynamics of the metal uptake in acanthocephalans is still missing. Usually, authors focused on studying the kinetics and metabolism of metals [3–5] or concentrated on the parasite’s accumulation capacity (reviewed in [1, 6]). However, a seasonal variation of metal concentrations in parasites exists as can be seen in a study on metal levels in the cestode *Bothriocephalus acheilognathi* [7], which is connected with the parasite’s transmission cycle during the year. The development of some fish acanthocephalans, especially the palaeacanthocephalans, is also characterised by typical annual infection patterns of intermediate and definitive hosts [8]. These patterns can be related to water temperature changes and are, therefore, season and climate dependent [9–11]. Moreover, the lifespan of most fish acanthocephalans in the intestine of the definitive host (in which metal uptake of the parasites occurs) does usually not exceed a period of some months [8]. Regarding the life-cycle of *Pomphorhynchus laevis* it is obvious that its development in the gut of the definitive host such as the barbel, *Barbus barbus*, is formally separated into two phases, which belong to different seasons of the year. The prepatent period begins immediately after the infection of the fish and is characterised by an enormous growth of the worms. It continues until the parasite is fully mature. The patent period covers the time from the release of eggs until the death of the acanthocephalan [12]. Therefore, seasonal analysis of metal concentrations in a host-parasite system would help to elucidate key aspects of the metal uptake process. For example, metal accumulation in host-parasite systems could be affected by the age structure of the parasite infrapopulation or by seasonal changes of host physiology during the year. The latter might be a result of differences in fish activity and metabolism during the cold season (e.g. winter). Changes in host activity are expected to have a considerable impact on the physiology of the parasite. Although only few data are available for longevity, fecundity and patency periods, it is known that fish acanthocephalans are able to survive seasonal periods of host starvation in contrast to acanthocephalans from homoeothermic hosts such as mammals [8].

All the above mentioned aspects have to be considered if acanthocephalans or other parasite taxa are taken as metal bioindicators. Additionally, the analysis of a seasonal metal accumulation dynamics could also be a good approach for a rough estimation of the lifespan of *P. laevis* in its definitive host. Such information is still missing as it is often impossible to examine the fish faeces qualitatively and quantitatively [8] in order to detect dead worms.

The aim of the present field study was to analyse a possible seasonal pattern of metal distribution in a host-parasite system in respect to the parasite’s degree of development in the gut of the definitive host. The freshwater cyprinid barbel (*Barbus barbus*) and its intestinal parasite *P. laevis* were selected as a model system, since they are already well known as a promising tool for environmental metal monitoring [13].

## Methods

### Ethics statement

Fish sampling was conducted by professional fishermen following the national laws and regulations of the Republic of Bulgaria.

### Fish sampling

Fish were collected in a seasonal manner (spring, summer, autumn) during the year 2006 from the River Danube near Kozloduy (Bulgaria; river kilometre 685). Sampling was performed during a project addressing the suitability of fish parasites as bioindicators (for additional information see Nachev et al. [2]). Fish were caught using gillnets and sacrificed by professional fishermen according to national laws and regulations. After sampling, fish were kept frozen until parasitological examination in the laboratory.

For the metal analyses eight barbels from each season were selected according to similar morphological characteristics such as body size and weight (see Table 1) as well as similar intensity of infection with acanthocephalans (82 ± 37 worms per fish). After dissection the total number of acanthocephalans was counted and the wet weight of each infrapopulation was recorded. The mean individual weight was calculated after dividing the total infrapopulation weight by the total number of worms. Acanthocephalans and the fish tissues (muscle, intestine and liver) were rinsed with double-distilled water and were kept frozen at -20 °C until metal analyses. To avoid contamination, all dissecting tools were previously cleaned with 1 % ammonium-EDTA solution (Titriplex® III, Merck, Darmstadt) and double-distilled water.

**Table 1:** Morphological data of *Barbus barbus* sampled during the year close to Kozloduy, Bulgaria

Parameter/ Season	Spring	Summer	Autumn
	(*n* = 8)	(*n* = 8)	(*n* = 8)
Weight ± SD^a^ (g)	430.9 ± 86.0	525.0 ± 243.0	373.8 ± 134.7
Total length ± SD (cm)	36.5 ± 2.7	37.4 ± 6.3	35.5 ± 7.2
Standard length ± SD (cm)	29.9 ± 2.2	31.1 ± 5.3	28.4 ± 4.2
Body height ± SD (cm)	7.2 ± 0.2	7.6 ± 1.2	6.9 ± 0.9
Condition factor ± SD	0.88 ± 0.09	0.95 ± 0.09	0.86 ± 0.22

^a^ *SD* standard deviation

### Element analysis

The samples were prepared for analysis using a microwave assisted digestion following the procedure described by Zimmermann et al. [14]. The weight of fish tissues (muscle, intestine and liver) taken for the digestion ranged between 150 and 340 mg (wet weight). The acanthocephalan weight used ranged between 20 to 130 mg (wet weight) due to expected higher metal levels. The parasite samples were previously pooled for individual fish and subsequently homogenised using a sonifier (Bandelin, Model Sonoplus HD2070). After digestion the clear sample solutions were analysed using inductively coupled plasma mass spectrometry (ICP-MS System Perkin Elmer- Elan 5000) and the concentrations of arsenic (As), cadmium (Cd), colbalt, (Co), copper (Cu), iron (Fe), manganese (Mn), molybdenum (Mo), nickel (Ni), lead (Pb), vanadium (V) and zinc (Zn) were determined (for details regarding instrumentation settings, calibration and sample measurements see Nachev et al. [2]).

The accuracy of the analytical procedure was verified by analysing dog fish muscle tissue (DORM–3, National Research Council, Canada) as certified reference material. Following analyses, the accuracy rates of seven certified elements were checked.

### Data analyses and statistical treatment

In order to compare metal concentrations between the host’s tissues and the parasites, arithmetic means with standard deviations were calculated. Additionally, the accumulation capacity of *P. laevis* was calculated as mean bioconcentration factors (BCF) for the different organs as described by Sures et al. [15] with the following equation: BCF = C _[*P. laevis*]_/C_[host tissue]_.

Wilcoxon matched pairs test was used to compare element concentrations in host tissues and parasites statistically. Differences in element concentrations and in acanthocephalan weight between the seasons were assessed with Kruskal-Wallis H-test. For possible correlations between the mean individual weight, the number of parasites and metal concentrations Spearman’s rank correlation was used.

### Element concentrations in the River Danube

The concentrations of the elements As, Cd, Cu, Pb and Zn in the water phase are shown in Table 2 [16]. The data is based on monthly monitoring (12 measurements) and represents two Danube sites: Novo Selo (km 845) situated 160 km upstream from our fish sampling locality and Iskar (km 637) located about 40 km downstream. As the mean concentrations determined during the years 2005 and 2006 at both Danube reaches were similar the concentrations at the site Kozloduy are unlikely to differ. The data also showed no clear signs for incidental contamination (hot spots) with these metals during the period 2005–2006.

**Table 2:** Element concentrations (μg/l) in water at two different sites along the Danube River in Bulgaria [16]

Element	Novo Selo (km 845 )		Iskar (km 637 )	
	2005	2006	2005	2006
As	2.2	2.275	2.692	2.382
Cd	nd	nd	nd^a^	nd
Cu	17.5	23.5	5.15	6.1
Pb	nd^a^	nd	2.767	2.364
Zn	22.0	20.92	29.73	20.0

*nd* element not detected; the concentration values were below the detection limits

^a^ Data refer to the second half of the year (6 measurements)

## Results

### Analytical procedure

The detection limits of the analysed elements as well as the concentrations obtained for the reference material (DORM–3) are listed in Table 3. The recovery rates of elements in dog fish muscle ranged between 87 and 106 %.

**Table 3:** Trace element concentrations in dogfish muscle certified reference material (DORM – 3) as well as accuracy and detection limits determined by ICP-MS analyses

Element	DORM3 certified ± SD (mg/kg)	DORM3 ICP-MS ± SD (mg/kg)	Accuracy (%)	Detection limit (μg/l)
As	6.88 ± 0.30	6.30 ± 0.40	92	0.008
Cd	0.290 ± 0.020	0.27 ± 0.02	94	0.01
Co	nc	–	–	0.01
Cu	15.5 ± 0.63	16.35 ± 0.93	105	0.19
Fe	347 ± 20	346.95 ± 28.24	100	2.76
Mn	nc	–	–	0.1
Mo	nc	–	–	0.02
Ni	1.28 ± 0.24	1.21 ± 0.15	94	0.47
Pb	0.395 ± 0.050	0.417 ± 0.043	106	0.26
V	nc	–	–	0.01
Zn	51.3 ± 3.1	44.4 ± 3.2	87	2.77

*nc* element not certified

### Element concentrations in fish tissues and parasite samples

The mean element concentrations in fish tissues and the parasites are presented in Table 4. The levels of As, Cd, Cu, Pb and Zn were found to be significantly higher in *P. laevis* than in the host tissues (Wilcoxon test, *Z* ranged from 2.54 to 4.37, *P* < 0.01). This trend was also clearly seen by the calculated mean bioconcentration factors for each tissue (Table 5). As expected, the lowest concentrations were observed in muscle tissue, which corresponds to the highest calculated BCF values. In general, the parasites demonstrated their enormous affinity to accumulate lead. The mean lead concentration in *P. laevis* determined for spring, for instance, was 1,194 times higher compared to that in muscle tissue and 78 and 211 times higher than in intestine and liver, respectively. Cadmium showed a similar pattern, but with lower bioconcentration factors (see Tables 4 and 5). Concerning the elements As, Cu and Zn, the concentration order differed slightly, with highest levels found in parasites followed by liver, intestine and muscle tissues. In summary, the acanthocephalans demonstrated an overall higher accumulation capacity compared to muscle tissue. For other organs such a clear difference was not obvious.

**Table 4:** Seasonal profile of mean (± SD) element concentrations (mg/kg) in different tissues of barbel and in *P. laevis*

Element/ Season	Tissue	Spring	Summer	Autumn
		(*n* = 8)	(*n* = 8)	(*n* = 8)
As	Muscle	0.28 ± 0.25	0.21 ± 0.15	0.19 ± 0.10
	Intestine	0.52 ± 0.32	0.35 ± 0.20	0.45 ± 0.17
	Liver	0.67 ± 0.42	0.54 ± 0.49	0.56 ± 0.21
	*P. laevis*	1.08 ± 0.54	1.01 ± 0.60	1.92 ± 1.23
Cd	Muscle	0.02 ± 0.03	0.01 ± 0.01	0.02 ± 0.01
	Intestine	0.24 ± 0.31	0.11 ± 0.07	0.14 ± 0.05
	Liver	0.16 ± 0.22	0.08 ± 0.04	0.16 ± 0.10
	*P. laevis*	2.40 ± 2.09	1.34 ± 0.55	2.63 ± 0.58
Co	Muscle	0.02 ± 0.01	0.02 ± 0.01	0.02 ± 0.01
	Intestine	0.15 ± 0.08	0.17 ± 0.14	0.19 ± 0.16
	Liver	0.04 ± 0.01	0.04 ± 0.01	0.05 ± 0.03
	*P. laevis*	0.07 ± 0.02	0.10 ± 0.07	0.13 ± 0.08
Cu	Muscle	1.45 ± 1.36	1.57 ± 1.29	1.00 ± 0.39
	Intestine	7.37 ± 4.87	4.79 ± 2.44	6.23 ± 2.85
	Liver	16.92 ± 11.59	13.40 ± 8.11	11.76 ± 5.90
	*P. laevis*	75.37 ± 35.43	53.13 ± 24.13	77.40 ± 35.84
Fe	Muscle	13.29 ± 3.54	10.21 ± 4.72	9.83 ± 4.10
	Intestine	84.55 ± 42.22	70.92 ± 17.87	118.19 ± 89.73
	Liver	71.88 ± 23.11	81.34 ± 39.42	67.94 ± 29.24
	*P. laevis*	76.62 ± 69.21	41.27 ± 23.28	37.59 ± 10.37
Mn	Muscle	0.37 ± 0.15	0.35 ± 0.11	0.45 ± 0.42
	Intestine	4.44 ± 1.48	7.35 ± 6.37	13.84 ± 14.64
	Liver	1.26 ± 0.33	1.49 ± 0.77	1.45 ± 0.80
	*P. laevis*	4.67 ± 0.85	7.64 ± 5.87	8.94 ± 6.87
Mo	Muscle	0.01 ± 0.004	nd	0.01 ± 0.01
	Intestine	0.06 ± 0.04	0.06 ± 0.02	0.03 ± 0.02
	Liver	0.21 ± 0.11	0.28 ± 0.16	0.13 ± 0.06
	*P. laevis*	0.05 ± 0.02	0.10 ± 0.06	0.05 ± 0.06
Ni	Muscle	1.26 ± 1.31	0.66 ± 0.61	0.31 ± 0.16
	Intestine	1.58 ± 0.74	2.28 ± 1.16	1.60 ± 0.84
	Liver	0.33 ± 0.22	0.21 ± 0.15	0.28 ± 0.15
	*P. laevis*	2.73 ± 1.30	0.55 ± 0.28	0.58 ± 0.26
Pb	Muscle	0.01 ± 0.01	0.004 ± 0.003	0.01 ± 0.01
	Intestine	0.09 ± 0.03	0.12 ± 0.10	0.48 ± 0.53
	Liver	0.03 ± 0.02	0.03 ± 0.02	0.06 ± 0.05
	*P. laevis*	6.83 ± 4.87	5.19 ± 3.74	9.81 ± 4.63
V	Muscle	0.04 ± 0.01	0.04 ± 0.01	0.03 ± 0.012
	Intestine	0.15 ± 0.05	0.29 ± 0.36	0.48 ± 0.398
	Liver	0.12 ± 0.05	0.17 ± 0.16	0.14 ± 0.094
	*P. laevis*	0.07 ± 0.01	0.10 ± 0.04	0.16 ± 0.069
Zn	Muscle	4.71 ± 1.49	4.76 ± 1.51	3.66 ± 0.69
	Intestine	11.39 ± 3.86	10.40 ± 1.16	10.14 ± 1.87
	Liver	18.82 ± 3.37	19.23 ± 5.84	18.00 ± 3.96
	*P. laevis*	63.91 ± 36.39	34.83 ± 16.66	91.06 ± 43.47

*nd* element not detected (concentration below the detection limit)

**Table 5:** Seasonal profile of bioconcentration factors (C _[*P.laevis*]_ / C _[barbel tissue]_) for *P. laevis* calculated with respect to different host tissues

Element/Season	Tissue	Spring	Summer	Autumn
As	Muscle	3.8	4.8	10.3
	Intestine	2.1	2.9	4.2
	Liver	1.6	1.9	3.4
Cd	Muscle	103.3	132.7	116.7
	Intestine	10.1	11.7	18.3
	Liver	15.3	16.0	16.8
Co	Muscle	4.4	5.7	8.3
	Intestine	0.5	0.6	0.7
	Liver	1.9	2.3	2.8
Cu	Muscle	50.7	33.9	77.2
	Intestine	10.0	11.1	12.4
	Liver	4.4	4.0	6.6
Fe	Muscle	5.8	4.0	3.8
	Intestine	0.9	0.6	0.3
	Liver	1.1	0.5	0.6
Mn	Muscle	12.8	21.9	20.0
	Intestine	1.1	1.0	0.6
	Liver	3.7	5.1	6.2
Mo	Muscle	7.0	5.9	5.0
	Intestine	0.8	1.6	1.2
	Liver	0.2	0.4	0.3
Ni	Muscle	2.2	0.8	1.9
	Intestine	1.7	0.2	0.4
	Liver	8.3	2.6	2.1
Pb	Muscle	1,194.4	1,250.0	794.4
	Intestine	78.1	42.2	20.2
	Liver	211.3	151.1	158.1
V	Muscle	2.0	2.8	5.2
	Intestine	0.5	0.3	0.3
	Liver	0.6	0.6	1.1
Zn	Muscle	13.6	7.3	24.9
	Intestine	5.6	3.3	9.0
	Liver	3.4	1.8	5.1

### Seasonal differences in acanthocephalan morphology

**Fig. 1:**
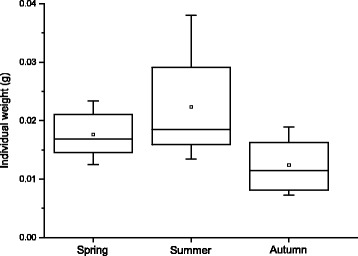
Seasonal profile of the mean acanthocephalan weight (*g*). Small squares in the boxes are medians, lines are means, boxes are interquartile ranges and error bars are interdecile ranges

The calculated mean individual weight for the parasite infrapopulations demonstrated a clear annual pattern (Fig. 1). The mean individual weight obtained in autumn was found to be significantly lower compared to summer (Kruskal-Wallis test; *H* = 6.797, *P* = 0.03), which suggests that the infrapopulations in autumn consisted mainly of young preadult individuals. The comparisons between spring and summer revealed no significant differences, still the acanthocephalan infrapopulations in summer showed a slightly elevated mean individual weight as expressed by the higher arithmetic mean and median values.

### Seasonal variation in accumulation of elements by *P. laevis*

**Fig. 2:**
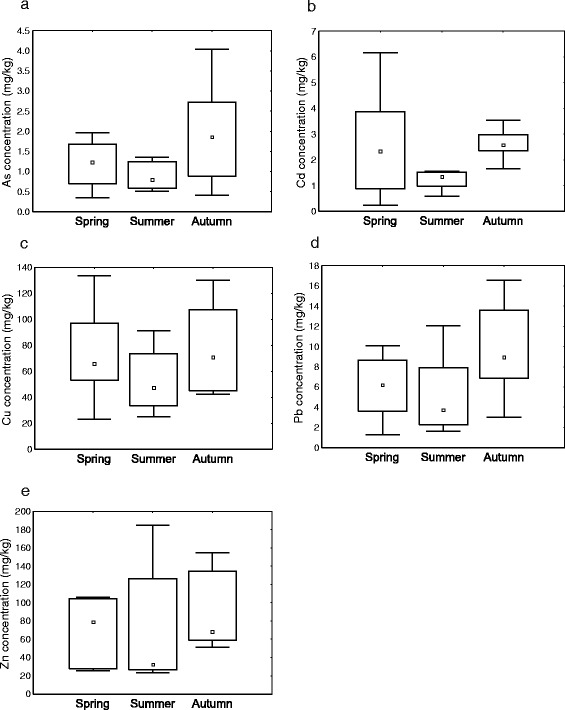
Seasonal pattern of the element concentrations determined for *P. laevis* (concentrations referred to wet weight). Small squares in the boxes are medians, boxes are interquartile ranges and error bars are interdecile ranges. **a** Arsenic (*As*). **b** Cadmium (*Cd*). **c** Copper (*Cu*). **d** Lead (*Pb*). **e** Zinc (*Zn*)

All elements (As, Cd, Cu, Pb, Zn) found in significantly higher concentration in the parasite demonstrated similar seasonal patterns (Fig. 2). Some metals showed a negative correlation with the calculated mean individual weight (e.g. Cd and Pb). Following the seasonal distribution of these elements, it turned out that lowest concentrations were detected in the acanthocephalans collected in the summer. On the other hand, the highest mean concentrations were obtained for autumn (see Table 4 and Fig. 2), whereas the only significant differences were found for cadmium comparing the levels in *P. laevis* between summer and autumn (Kruskal-Wallis test; *H* = 6.965, *P* = 0.03).

As mentioned above, the concentration of some elements (e.g. Cd and Pb), which were accumulated by the acanthocephalans to a higher degree, showed a clear relation to the mean individual weight of acanthocephalans. In general, a higher mean individual weight corresponded to a lower load of cadmium (Spearman’s rho = -0.47, *P* < 0.05) and lead (Spearman’s rho = -0.43, *P* < 0.05). Such a correlation was not found to be statistically significant for As and the essential elements Cu and Zn.

## Discussion

Our results for element concentrations in the host-parasite system are in line with other studies and confirm accumulation patterns described earlier for acanthocephalans and their fish hosts. For example Schludermann et al. [17] found considerably higher concentrations of Cd, Pb and Zn in *P. laevis* compared to the levels in barbel tissues in fish caught in the Austrian part of the River Danube. Similar results were also published by Thielen et al. [18] for fish from the River Danube near Budapest after analysing a wide number of elements. Apart from barbel, *P. laevis* showed an enormous accumulation capacity (e.g. for lead) also in other fish species such as chub (*Squalius cephalus*) [19]. According to the data available, there is no doubt that the acanthocephalans are very useful in terms of metal indication, due to their excellent response to ambient element levels.

The observed seasonal pattern of metal concentrations in the parasites was suggested not to be a result of an incidental contamination (hotspot pollution) in the respective part of the river. Aqueous concentrations of the investigated elements at two sites (upstream and downstream from our sample location) showed no considerable variation over the months in the period 2005–2006 (see Table 2). Therefore, different body burdens during the year can probably be explained by the seasonality of acanthocephalan transmission, i.e. the degree of development (maturation) in the gut of the final host [10] and/or changes in the activity, feeding behaviour and physiology of the latter [20]. Seasonal aspects in the transmission were reported for various aquatic parasites, with climate conditions playing the decisive role. Moravec & Scholz [11] observed seasonality in the occurrence and maturation of the acanthocephalan *Neoechinorhynchus rutili* in barbel. Interestingly, the seasonal pattern was in accordance with that observed for *P. laevis* in the present study. The variation in prevalence of acanthocephalans in intermediate and final hosts was assumed to be a consequence of seasonal fluctuation in the temperature of the habitat [9–11]. For instance barbel’s activity differs strongly in terms of water temperature, as it decreases progressively with the decrease of water temperature until reaching its thermal limit for activity at 4 ° C (dormancy phase) [20]. The geographical area where the present barbel sampling was conducted is characterised by a typical continental climate and therefore the water temperature in winter months (December to March) is mostly below or around the barbel’s thermal limit of activity [16]. As a result, the reduced fish activity and altered feeding behaviour probably leads to a lack of infection during winter months, as the final host stops feeding on intermediate hosts, gammarids. With rising temperatures, the barbel starts feeding again, but an increase in new parasite infection occurs in late summer, when more infected intermediate hosts are available. The low temperatures may affect feeding ​behaviour and reproductive activity of the amphipods as well [21]. Therefore, a pronounced seasonality of infection with cystacanths could be expected, as has been already observed by many authors (e.g. Molloy et al. [22]; summarised by Kennedy [8]).

**Fig. 3:**
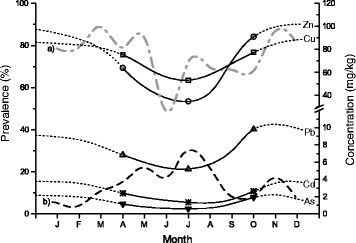
Seasonal pattern of the concentrations of the elements As, Cd, Cu, Pb and Zn in adult *Pomphorhynchus laevis* fitted according to changes in prevalence of **a** adult *P. laevis* in fish and **b** cystacanths in gammarids [23]. (From Kennedy [8, 10])

Finally, the combination of the available literature data [8, 10, 23] and the chemical data acquired in the present study suggests that the seasonal pattern of metal accumulation corresponds most likely to the seasonal dynamics of acanthocephalan transmission (Fig. 3). Due to the seasonality of infection in the final host, it could be expected that the age composition of acanthocephalan infrapopulations changed during the seasons. The differences obtained for the worms’ mean weight in the course of the year (see Fig. 1) could be taken as evidence for this assumption. Simultaneously, the concentration patterns for all elements were similar throughout the year, which was an additional sign that metal uptake (independent of the type of metal) was probably related to the stage of development. Thus, following the development of acanthocephalans with respect to the accumulation process it seems that in autumn *P. laevis* infrapopulations consisted mainly of young worms, which occurred in their growth phase (prepatent period). Therefore, due to accelerated metabolic processes the mean concentrations of the accumulated elements were the highest. The negative relationship between the concentrations of the elements Cd and Pb and the mean individual weight additionally explained the relationship between the accumulation process and the degree of development (characterised by the mean individual weight). A similar tendency was described for other organisms established in metal monitoring like shellfish, for which smaller individuals are usually characterised by faster uptake than the larger ones [24]. Increased surface-volume ratios of younger (smaller) specimens lead to a higher uptake from the water and causes negative relationships between size and concentration. The same could be true for fish acanthocephalans, as the assimilation of nutrients and metals occurs mainly through the worm’s tegument, which supports the importance of a surface to volume ratio also for acanthocephalans.

For winter and early spring, it could be expected that the down-regulated metabolism of barbel during the cold months affects the metabolism of acanthocephalans, including their metal uptake capacity. This leads to a decrease of metal concentrations in spring compared to autumn. In the period of dormancy, a process of metal elimination might appear as well. These aspects combined with the growth factor during the autumn, led possibly to a decrease in element concentrations. If the tissue grows faster than metal uptake occurs, concentrations in parasite tissue may be diluted as described by Strong & Luoma [24] for free living sentinels. This is most likely one of the major reasons for the deviation of metal concentration in the autumn-spring period.

During late spring and early summer the mean individual weight slightly increased, which suggests that almost all individuals reached the adult stage at which they reproduce and subsequently die. Accordingly, the mean metal concentrations in the parasites continue to decrease, which indicates that the accumulation process mainly takes place during the prepatent period (in autumn). Our accumulation data suggest that the acanthocephalans have reached the element equilibrium concentration at the beginning of the patent period when metal accumulation is (at least) compensated by elimination processes [19]. Active excretion of elements with the eggs released by females during the reproduction period might be an additional factor during the metal elimination process, as Sures et al. [25] reported that acanthocephalans are able to discharge metals via the shells of their eggs. This assumption was proven by higher Pb concentrations in eggs in comparison to the worm’s body wall and host tissues [25]. This kind of detoxification mechanism probably appears not only to be true for the archiacanthocephalan *Moniliformis moniliforms*, for which it was first described, but also for palaeacanthocephalans, to which *P. laevis* belongs. Due to the similarity in the concentration patterns of the elements investigated here, it appears likely that other metals are also excreted via egg shells.

**Fig. 4:**
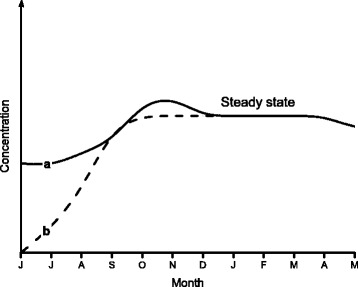
Model of metal accumulation in *P. laevis* derived from present data **a** and a generally accepted metal uptake model [26] **b** considering data from laboratory studies [5, 19]

Based on the present data and laboratory data from chub infected with *P. laevis* [5, 19] a model can be suggested. It visualises the accumulation kinetics of metals and As under natural conditions (Fig. 4). This model considers a generally accepted metal uptake kinetics [26], and similarly combines laboratory uptake data of lead [5,19] as well as the specific annual reproduction and infection cycle of *P. laevis* under the local climate conditions. Slight deviations between field and laboratory accumulation kinetics can be attributed to the time of infection, which can last over a range of some weeks during the warm months, whereas in laboratory studies uninfected fish were experimentally infected at the same time (for details see [19]). The natural infection process reduces the homogeneity of acanthocephalan infrapopulations as not all individuals are in the same developmental stage and are therefore not exposed to metals for the same period of time. Accordingly, there is a shift in the accumulation process when comparing the initial metal concentrations (see Fig. 4). Overshooting metal concentrations between October and December are caused by an accelerated metabolism of young acanthocephalans during their growth phase immediately after establishing in the definitive host’s intestine. Subsequently, the level decreases to a steady state concentration during winter and spring. This slightly lower steady state level results from reduced host activity during winter month. After the acanthocephalans matured in early spring they start reproducing during April to June. Therefore, our model considers a decrease in element concentrations, which was related to the metal elimination via egg release. A synthesis of the annual course of element concentrations and the natural infection process allows predicting the lifespan of *P. laevis* to be approximately 7–8 months.

## Conclusions

In order to use organisms as biomonitors and to identify the appropriate period for sampling the seasonal effects on metal concentration in the respective monitor organism have to be elucidated in detail. Seasonal variations of metal uptake and accumulation which are mostly associated with the reproductive cycle and growth are well known for established sentinels such as bivalves [27]. After the release of gametes bivalves for example have depleted their energy reserves and thus accelerate their metabolism which can also be seen in increased metal uptake. Additionally, aspects such as body size might affect the metal accumulation, whereas smaller (younger) individuals show a higher accumulation activity than bigger (older) specimens as demonstrated by Wang & Fisher [28]. When drawing parallels between free living sentinels and parasites it appears that fish acanthocephalans bear some advantages if they are taken as metal monitors. Even the fact that fish are randomly infected during their active periods and thus have a heterogeneous acanthocephalan infrapopulation does not prevent the use of parasites as monitor organisms for metal pollution, as their metal concentrations remain rather similar in autumn, spring and probably in winter months. Therefore, there is quite a long period for acanthocephalan sampling compared to other free-living sentinels. However, in order to reduce variation during long term studies it would be advantageous if sampling was performed at least in the same season. If highest accumulation rates are necessary, e.g. to detect even lowest aqueous pollution levels, acanthocephalan sampling should be performed in late autumn or early winter.

## Abbreviations

BCF: bioconcentration factors
ICP-MS: inductively coupled plasma mass spectrometry
